# Insuffisance cardiaque aiguё congestive due au Bevaczumab dans le cancer du sein métastatique: à propos d’un cas

**DOI:** 10.11604/pamj.2022.43.50.34743

**Published:** 2022-09-30

**Authors:** Gloria Akimana, Remy Onana, Siham Lkhooyali, Hassan Errihani

**Affiliations:** 1 Service d’Oncologie Médicale, Institut National d’Oncologie, Rabat, Maroc

**Keywords:** Sein métastatique, Bevacizumab, insuffisance cardiaque congestive, cas clinique, Metastatic breast, Bevacizumab, congestive heart failure, a case report

## Abstract

L´adjonction du Bevacizumab à la chimiothérapie a montré son intérêt en première ligne dans le cancer du sein métastatique en améliorant la survie sans progression (SSP) mais pas la survie globale (SG). Après un certain délai d´exposition l´apparition d´effets secondaires telle que l´Insuffisance cardiaque congestive (ICC) aiguё a été décrite au cours des essais cliniques, se manifestant par une baisse de la fraction d´éjection du ventricule gauche (FEVG) et des signes cliniques ayant parfois occasionnée une hospitalisation. Nous rapportons le cas d´une patiente chez qui ce délai d´exposition a été plutôt long avec une très longue survie sous Bevacizumab. La survenue de l´hypertension artérielle est apparue au bout de 8 mois de traitement par Bevacizumab en association avec une chimiothérapie en première ligne métastatique, tandis que l´ICC aiguё est survenue après 10 ans de traitement environs soit à la 129^e^ cure. L´évolution de l´ICC a été favorable après un traitement adéquat et arrêt du Bevacizumab comme dans la plupart des cas rapportés dans la littérature.

## Abstract (en)

The addition of Bevacizumab to chemotherapy has shown its first-line interest in metastatic breast cancer by improving PFS but not OS. After a certain period of exposure, the appearance of side effects such as acute Congestive heart failure (CHF) has been described during clinical trials; manifested by a drop in LVEF and clinical signs which sometimes caused a hospitalization. We report the case of a patient in whom this exposure time was rather long with a very long survival under Bevacizumab. The onset of arterial hypertension appeared after 8 months of treatment with Bevacizumab in combination with metastatic first- line chemotherapy, while acute CHF occurred after approximately 10 years of treatment, which is at the 129^th^ treatment. The evolution of CHF was favorable after adequate treatment and discontinuation of Bevacizumab as in most cases reported in the literature.

## Introduction

**Figure 1:**
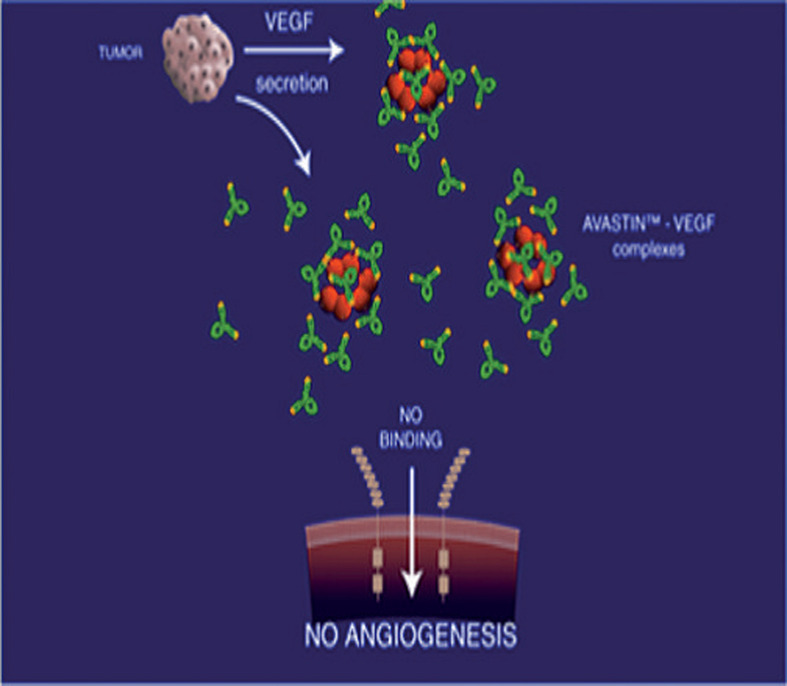
mode d´action du Bevacizumab

Le Bevacizumab est un anti-angiogénique anti-*Vascular Endothelial Growth Factor (VEGF)*, qui a montré son efficacité en première ligne dans le traitement du cancer du sein métastatique en association à la chimiothérapie avec une amélioration significative de la SSP (Figure 1). Néanmoins le Bevacizumab n´est pas dénué d´effets indésirables car il peut être responsable notamment de l´HTA, d´une protéinurie pouvant aller jusqu´à un syndrome néphrotique, de l´augmentation du risque thromboembolique [1]. Un risque d´insuffisance cardiaque congestive est également rapporté dans les essais cliniques à des taux variables mais faibles. La détection des effets secondaires des traitements anti cancéreux constitue souvent un des grands défis dans la prise en charge des patients atteint de cancer. En effet, ils peuvent être à l´origine des arrêts de traitement pour cause d´intolérance sans qu´il y ait eu progression. L´objectif de cette étude est de rapporter le cas d´une insuffisance cardiaque congestive survenue chez une patiente après plus de 10 ans de traitement.

## Patient et observation

**Information de la patiente:** patiente âgée de 58 ans, sans antécédents particuliers ni risques notamment cardiovasculaires, qui était traitée depuis 2004 pour un cancer du sein droit.

**Résultats cliniques:** à l´examen physique on retrouve une performance status à 0, une zone de mastectomie propre, sans autre particularités par ailleurs.

**Démarche diagnostique:** en 2004 la patiente avait bénéficié d´une mastectomie puis chimiothérapie, radiothérapie et hormonothérapie en adjuvant pour son cancer du sein qui était localisé. En 2008 elle a fait une rechute métastatique agressive au niveau osseux, RH+Her2-. Après réunion de concertation pluridisciplinaire la décision a été d´instaurer un traitement à base de Bevacizumab à la dose de 10mg/kg en association avec le Paclitaxel, avec une évaluation tous les trois mois et contrôle de la TA et de la protéinurie avant chaque cure. L´évolution a été très satisfaisante avec une nette amélioration clinique, réponse radiologique presque complète et une bonne tolérance du traitement et donc le traitement a été maintenu sous surveillance.

Au bout de 8 mois de traitement soit 10 cures elle a développé une Hypertension artérielle (HTA) (17/9,5cmHG), découverte lors de l´examen clinique de routine. Une consultation chez le cardiologue a été faite qui a confirmé l´hypertension artérielle. Ensuite à la 129^e^ cure elle a présenté un syndrome d´anasarque, le bilan biologique fait (Bilan rénal, hépatique entre autres) était normal avec une protéinurie négative. Une consultation et un bilan en cardiologie. Une échographie cardiaque a été réalisée et a révélé une poussée d´insuffisance cardiaque avec FEVG à 25% mais TA normale.

**Intervention thérapeutique et suivi:** lors de la découverte de l´HTA, un traitement a été initié par les inhibiteurs calciques avec stabilisation de la tension artérielle. Ses chiffres tensionnels ont par la suite été fluctuants entre 13/7cmHg et 15/8cmHg. Après survenue de l´ICC aiguё, elle a été mise sous traitement à base de diurétiques, Inhibiteurs calciques, IEC, bétabloquants, ARAII, digitaliques. Sous cette combinaison de molécules l´évolution a été bonne, avec une FEVG de contrôle à 60%. Après la survenue de cette insuffisance cardiaque congestive la décision a été d´arrêter définitivement le Bevacizumab en continuant le traitement hormonal débuté entre temps.

**Consentement éclairé:** un consentement éclairé écrit, daté et signé a été obtenu du patient ayant permis la réalisation de ladite exploration.

## Discussion

Le cancer du sein est le plus fréquent et le plus meurtrier chez la femme. Son pronostic est d´autant plus péjoratif qu´il est métastatique, par rapport au cancer du sein localisé, avec une médiane de survie de 2 à 3 ans [2]. Sa prise en charge a beaucoup évolué ces dernières années avec l´apparition de thérapies ciblées notamment les anti-angiogéniques tel que le Bevacizumab. C´est un AC monoclonal humanisé dirigé contre l´angiogenèse tumorale par blocage du VEGF auquel il se lie pour empêcher sa liaison à son récepteur spécifique. Il y a ainsi diminution des vaisseaux sanguins et régression de la croissance tumorale [3]. Des effets secondaires vont toutes fois se manifester, les plus fréquents étant à type de survenue et/ou accentuation d´une HTA, protéinurie, accidents thromboemboliques, accidents hémorragiques, etc. [1].

Trois essais de phase III (E2100, AVADO et RIBON-1) ont mis en évidence l´intérêt du Bevacizumab en association a la chimiothérapie en première ligne, notamment avec amélioration de la SSP (11,8 mois vs 5,9 mois pour E2100) et de la RO (36,9% vs 21,2% dans E2100) sans amélioration significative de la survie globale (26,7 mois vs. 25,2 mois dans E2100) [4-6]. La durée de ce bénéfice pour les trois études variait entre 0,7 et 5,6 mois [7].

Dans ces études, ainsi que dans une étude de phase IV ATHENA M031 qui a évalué la tolérance du Bevacizumab en association avec une chimiothérapie en 1^e^ ligne avec poursuite du Bevacizumab jusqu´à progression ou intolérance [8], une baisse de la fraction d´éjection ventriculaire a été rapportée dans 0-2,9% des cas [7]. Dans la littérature l´ICC a été observée dans toutes les études menées avec Bevacizumab et principalement chez les femmes suivies pour cancer du sein métastatique. En effet, dans l´étude AVF2119g [9] une augmentation de l´incidence de l´ICC (3.5 VS 1%) a été observée chez des patientes ayant reçu un traitement antérieur par anthracyclines, une radiothérapie sur la paroi thoracique gauche, ou ayant un FDR de survenue d´une ICC. Dans la même étude, comme pour notre patiente, l´ICC s´est manifestée par une simple baisse de la FEVG asymptomatique ou par des signes d´ICC ayant nécessité un traitement ou une hospitalisation [9].

Chez notre patiente, le délai d´apparition de la toxicité cardiaque a été de 8 mois pour l´HTA et de 10 ans environ pour l´ICC, soit 129 cures. Ce délais est largement supérieur à celui retrouvé dans la littérature: une moyenne de 9 cures pour un nombre maximum de 29 cures dans une étude rétrospective sur 2 ans avec comme motifs principaux d´abandon du traitement la progression et la toxicité [10] et une médiane de 15 cures (3-34) dans une autre étude marocaine [11]. Une autre étude descriptive des patientes longues survivantes au Bevacizumab a rapporté un taux d´ICC de 1,3% après une médiane de suivi de 33 mois [12]. En général une évolution favorable après un traitement adéquat a été notée chez les patientes ayant manifesté une ICC sous Bevacizumab dans la littérature [9] comme chez notre patiente.

## Conclusion

L´insuffisance cardiaque congestive chez cette patiente sans facteurs de risques cardio-vasculaires a été associée à la toxicité cardiaque du Bevacizumab. Des cas similaires sont rapportés dans les essais cliniques mais celui-ci est à notre connaissance le premier rapporté chez une patiente très longue répondeuse au Bevacizumab en pratique clinique. L´évolution sous traitement adéquat a été favorable.
